# Pregnant women’s views on the acceptability, enablers, and barriers of participation in a randomized controlled trial of maternal posture for fetal malposition in labor

**DOI:** 10.18332/ejm/144057

**Published:** 2022-01-28

**Authors:** Jennifer Barrowclough, Bridget Kool, Caroline A. Crowther

**Affiliations:** 1 Liggins Institute, The University of Auckland, Auckland, New Zealand; 2 Section of Epidemiology and Biostatics, School of Population Health, Faculty of Medical and Health Sciences, University of Auckland, Auckland, New Zealand

**Keywords:** survey, randomized controlled trial, pregnant women, posture, labor presentation

## Abstract

**INTRODUCTION:**

Fetal malposition in labor results in adverse maternal and infant health. Whilst evidence for effective interventions is inconclusive, based on the hypothesis that gravity corrects malposition, the feasibility and design of a randomized controlled trial (RCT) to improve maternal and infant health outcomes should be considered. The aim was to assess pregnant women’s views on the acceptability, enablers, and barriers of participation in an RCT of maternal posture for fetal malposition in labor.

**METHODS:**

A web-based anonymous survey of pregnant women was conducted in Auckland during 2020. Quantitative data were summarized descriptively using a chi-squared test to assess differences in proportions. Maternal characteristics influence on women’s responses was assessed using cross-tabulation. A thematic content analysis of free text responses was undertaken.

**RESULTS:**

Most of the 206 respondents were aged 26–35 years (75%), 29–38 weeks pregnant (71%), of European (40%) or Asian (36%) ethnicity, and similarly nulliparous or multiparous. Most women (76%) knew of fetal malposition in labor; however, only 28% were aware of maternal posture to correct this. Most women (86%) were interested in labor research and although 37% would participate in an RCT, almost half (47%) were unsure and a 15% would not participate. Concerns mostly related to comfort (22%). Nearly half of women (49%) would need to consult their partner regarding participation in an RCT.

**CONCLUSIONS:**

Enablers for participation in a posture trial in labor include measures to enhance maternal comfort, increasing awareness of malposition and the role of posture, and involving partners in pre-trial counselling and recruitment.

## INTRODUCTION

Approximately one-quarter of all women in labor experience fetal malposition^1^. Women with a fetal malposition have an increased risk of prolonged labor^2,3^, oxytocin augmentation, use of epidural^2,4-7^ instrumental vaginal birth, serious perineal trauma, caesarean section^2,3,5,6^, and postpartum hemorrhage related to caesarean birth^2,3,7,8^. In addition, malposition has an emotional impact on some women, for example, a narrative study describing ‘conversations’ with 50 women revealed women’s anger at not being told of the fetal position in labor, therefore missing the opportunity to apply gravity to posture; the sense of loss from caesarean section; and stress from knowing there is a malposition^9^. Infants of persistent malposition are more likely to be admitted to a neonatal intensive care unit (NICU), and experience birth injury^10-12^. Several randomized controlled trials (RCTs) of maternal posture assess the safety and effectiveness of interventions to improve maternal and infant outcomes of fetal malposition^13-22^. However, the efficacy of maternal posture to correct malposition remains inconclusive indicating further RCTs are needed. Some studies have assessed women’s views during a trial or retrospectively, on the comfort and acceptability of the hands and knees posture^6,13,14^. However, little is known of women’s understanding of fetal malposition or their views on postures in labor for correcting malposition. This information could identify factors influencing women’s participation in studies of maternal postures for malposition and inform the feasibility and design of any future RCT.

Enablers and barriers for participation in RCTs, that were reported in a recent Cochrane systematic review, relate to methods of recruitment, expectations of improved health, incentives, the burden of appointments, confusion between current care and future care, and the influence of care givers and family^23^. The review included a qualitative study using interviews with 10 pregnant women who had participated in an RCT of the hands and knees posture twice daily from 37 weeks gestation until birth, to assess why they participated and what influenced their compliance with the intervention. Only a quarter of women carried out the exercises for various reasons including discomfort and forgetfulness. However, the study revealed women highly valued midwifery-led research and believed midwives best understood labor and birth^24^. The chance of improved health outcomes for the infant has been identified as the most likely reason for trial participation^24,25^ and implies that the women assumed the intervention was better than the control. Relationships built on partnership and advocacy, found in the New Zealand Midwifery Scope of Practice^26^, may lead to the midwife having an influence over a woman’s decision to participate in a research trial during labor. A recent retrospective review reported a higher rate of participation in a trial of manual rotation for women with fetal malposition during labor rather than in pregnancy^27^.

The Cochrane review that assessed the use of posture in late pregnancy or in labor for fetal malposition found that the hands and knees position did not reduce the risk of a caesarean birth, however back pain in labor was reduced^28^. The effectiveness of RCTs including this and other postures in labor on fetal malposition is being assessed in an update of the Cochrane review^29^. Whether use of maternal posture improves health outcomes following malposition in labor is unclear, including whether women consider such postures acceptable to use in labor.

The aims of this study were therefore to assess pregnant women’s awareness of fetal malposition and use of maternal posture to correct malposition; their views concerning use of the Sims posture in labor; the acceptability of participation in an RCT of maternal posture for fetal malposition in labor; and the need for their partner to be involved in the decision to participate in an RCT.

## METHODS

An anonymous web-based survey was conducted during 2020 (January–November) amongst pregnant women in the Auckland Hospital region. The region provides primary, secondary, and tertiary care to 6500 women annually from one of four public hospitals in Auckland, a contracted private birthing unit, and midwives providing home births. Women needed to be aged ≥16 years and receiving care within the Auckland Hospital region to be eligible to take part in the survey. Māori [New Zealand’s indigenous population] pregnant women comprise around 7% of women cared for by Auckland Hospital’s maternity services^30^, however, Māori comprise 16% of the national population^31^. Therefore, attempts were made to over sample pregnant Māori women by widening their inclusion from at least 24 weeks’ gestation, whilst pregnant non-Māori women were included from at least 28 weeks’ gestation.

The survey was advertised via brochures and QR coded posters in hospital and community clinics, including public and private midwifery and obstetric care settings, and as email attachments for some virtual clinics and antenatal classes. Paper copies of the surveys were available and could be submitted via a drop box located in the clinics. Midwives were encouraged to share the study brochure with their clients. In addition, the lead researcher (JB) discussed the study with pregnant women attending the hospital’s antenatal clinics (low risk and diabetes clinics). Follow-up with midwives was made at monthly meetings to seek feedback on recruitment and provide additional brochures/posters as required. COVID-19 pandemic priorities impacted on recruitment and, therefore, the survey was available to access for 10 months instead of the planned two months.

A sample size of 215 was estimated based on the likelihood that a minimum of 50% of the views expressed by women in the sample reflect the wider Auckland pregnant population’s views with a 95% confidence level and a margin of error ± 6 based on the hospital’s bimonthly birth rate of 1135 in 2019^30^ using Calculator.net software.

The survey was created and delivered using Qualtrics software (Qualtrics, Provo, UT). The questionnaire was piloted with a small number of pregnant women to check functionality and comprehension of the questions, and revised accordingly. The survey comprised 15 questions. Areas of interest included: respondent background (parity, gestation, maternal age, ethnicity, and previous experience of malposition in labor); interest in labor research; acceptability of the illustrated Sims posture (including any cultural, religious, comfort, safety, or other concerns); willingness to participate in a future RCT (allocation explained as random); and desire to consult their partner before consenting to participate. Ethnicity was collected based on HISO 10001:2017 Stats NZ level 2 criteria^32^ with options to select as many as apply and was therefore prioritized into Stats NZ level 1 criteria^32^ including Māori, Pacific Peoples, European, Asian, Middle Eastern, Latin American, African [MELAA] and Other.

Quantitative data were described as frequency and percentage, and analyzed descriptively using chi-squared statistics in SPSS (SPSS for Windows version 27, SPSS Inc., Armonk, NY, USA). Fisher’s exact test was used when cells had values less than 5. To determine the influence of a respondent’s characteristics on the acceptability of the Sims posture, and participation in a future RCT, cross tabulation was performed using chi-squared.

A thematic content analysis was applied to qualitative data from free text responses using Trello software (Trello Inc., Atlassian). Quotes to illustrate themes are identified by a unique study ID, age, and ethnicity. Coding of data was agreed using a second non-midwife reviewer resulting in some minor revisions.

## RESULTS

Of the 210 surveys completed, four respondents were ineligible due to maternal age or gestational age leaving a total of 206 respondents.

### Maternal characteristics

Most women were aged 26–35 years (75%), between 29–38 weeks pregnant (71%), of European (40%) or Asian (36%) ethnicity and had no previous experience of fetal malposition in labor (88%) (Table 1); 8% of women identified as Māori. There were similar numbers of nulliparous and multiparous women, 52% versus 49%.

**Table 1:** Maternal characteristics of survey respondents in Auckland, New Zealand 2020 (N=206)

*Characteristics*	*Total*	*n (%)*	*p*
**Age** (years)	178		<0.001
16–25		11 (6.2)	
26–35		134 (75.3)	
≥36		33 (18.5)	
**Ethnicity**	176		<0.001
Māori		14 (8.0)	
Pacific people		24 (13.6)	
European		70 (39.8)	
Asian		63 (35.8)	
MELAA^a^		5 (2.8)	
**Parity**	204		0.674
0		105 (51.5)	
≥1		99 (48.5)	
**Gestation** (weeks)	175		<0.001
24–28		38 (21.7)	
29–33		56 (32.0)	
34–38		69 (39.4)	
≥39		12 (6.9)	
**Previous malposition**	205		<0.001
Yes		26 (12.7)	
No		179 (87.3)	

a Middle Eastern, Latin American, African.

### Knowledge of malposition

**Figure 1:**
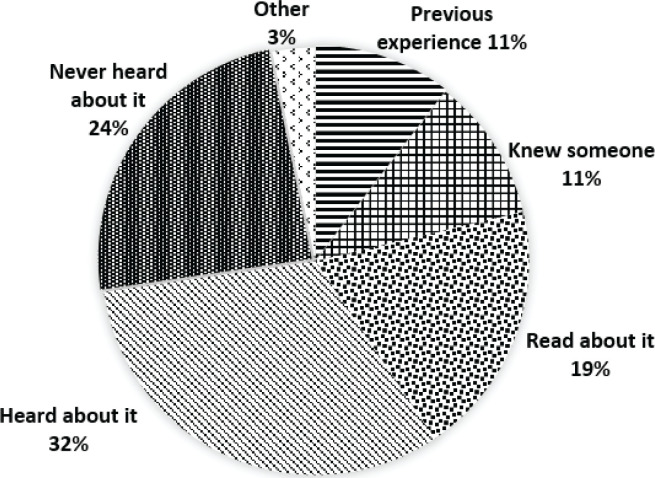
Respondents’ source of knowledge of fetal malposition in Auckland, New Zealand during 2020 (n=201)

A quarter of multiparous women had experienced fetal malposition in labor (n=26; 26%) (Table 1). However, the majority were aware of malposition (76%) mostly from other sources (65%) (knew someone who had it, read/heard about it, or other) (Figure 1). Those who had never heard of malposition were mostly aged 26–35 years (63.8%, p=0.012), and whilst more women were nulliparous than multiparous (55% vs 45%) this difference was not significant (p=0.459). The majority of women (n=128; 63%) had not heard of using posture in labor to correct malposition. For the 57 (28%) women who had heard of the approach, their sources of knowledge included online (n=22), midwife (n=17), doula/childbirth educator/antenatal class (n=15), magazine/book (n=12), family/friend (n=6), other (n=6), and obstetrician (n=4).

Free text responses regarding previous level of knowledge about malposition, received from seven respondents (three of whom were health professionals) revealed personal/current experience, and views that birth was difficult/complicated, for example:

*‘I heard it is more difficult to give birth and mum may need a c-section.’* (P129, age 26–35 years, Asian)

Among the 26 women who had a previous labor with a fetal malposition, similar numbers of women were satisfied (n=12) or not satisfied (n=10) with their labor, and emotionally positive (n=9) or negative (n=10) about their experience. Four women were neutral regarding their satisfaction, and seven neutral regarding their emotional experience.

### Acceptability of the Sims posture

Just over half of women viewed the Sims posture as acceptable (n=93; 52%) compared to being not sure or having concerns, and had no concerns regarding comfort, cultural, religious, safety or other concerns.

Free text comments concerning the Sims posture were provided by women in relation to overall acceptability (n=25), comfort (n=38), safety (n=14) and other concerns (n=3).

Themes relating to the overall acceptability of the Sims posture included: need for free mobility, spread of epidural, time in position, acceptable ‘if it works’, comfort and safety.

Subthemes related to ‘comfort’ included: time in posture, pain, prefer standing or sitting, need to move, willing to try, and easier breathing in the posture.

‘Safety concern’ subthemes included: seeking reassurance that it is safe, squashing the fetus, reducing blood supply, fetal monitoring difficulty, risks of pressure sores, and querying ligament and nerve irritation.

Other concerns regarding the Sims posture included: causing tiredness, difficulty performing vaginal examinations, and use of pillow between knees as awkward.

### Influence of maternal characteristics on acceptability of Sims posture

Whether a woman considered the Sims posture acceptable was influenced by maternal age, ethnicity, and whether they had heard of fetal malposition and the use of maternal posture for fetal malposition, but was not influenced by any other maternal characteristics.

Women aged ≥26 years were most likely to consider the Sims posture as acceptable (55%) compared to women aged 16–25 years who were mostly unsure whether the posture was acceptable (82%) (p=0.010).

Women were more likely to be unsure about the acceptability of the Sims posture (53%) if they were unaware of fetal malposition compared to those who were aware of it and mostly thought it acceptable (55%) (p=0.001). Restricted movement was the predominant theme for women who were aware of malposition and thought the posture unacceptable. Women who were unaware of fetal malposition were more likely to be unsure whether they had cultural concerns (p=0.026), religious concerns (p=0.015), or comfort concerns (p<0.001) related to the posture, compared to women who were aware of fetal malposition.

Similarly, women were more likely to be unsure about the acceptability of the Sims posture if they were unaware of using maternal posture for fetal malposition, compared to women who were aware of using posture (41% vs 18%) (p=0.010). MELAA, European, and Asian women were more likely to indicate the posture was acceptable (100%, 61%, and 51%, respectively), compared to Māori and Pacific women who were more likely to be unsure (43% and 63%, respectively) (p<0.001). Those who considered the posture unacceptable were mostly European (n=17; 24%) or Māori (n=3; 21%).

Women of all ethnicities were most likely to have no cultural concerns regarding the Sims posture (71–96%), followed by not sure (4–21%). European and Māori were the ethnicities most likely to have some comfort concerns regarding the Sims posture (41% and 29%, respectively) compared to other ethnicities (7%).

### Participation in an RCT of posture for malposition

**Figure 2:**
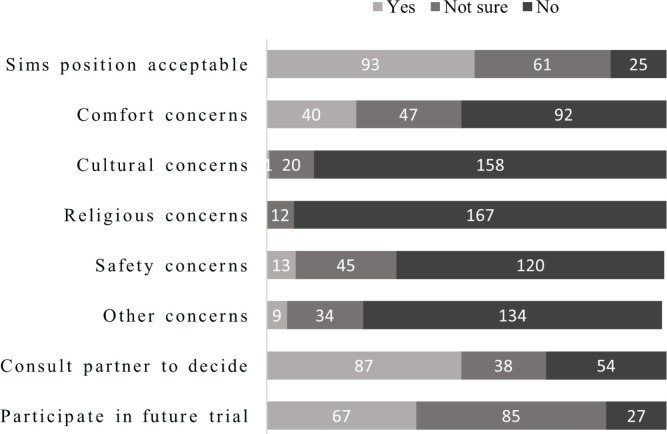
Respondents' views on the acceptability of an RCT of maternal posture for fetal malposition in labour, in Auckland, New Zealand during 2020 (n=179)

The majority of women were interested (n=93; 52%) or very interested (n=60; 33.5%) in labor research. However, only 37% (n=67) of women indicated they would participate in a trial of maternal posture for fetal malposition in labor (Figure 2). Nearly half of the women (n=85; 48%) selected ‘maybe’ in response to the question on participation. A similar proportion of women indicated they would want to consult their partner before deciding to participate in a trial (n=87; 49%).

Free text comments (n=17) from those not willing to participate in a future RCT (n=27) included the themes: freedom within the control group to use the intervention posture; wanting to be in the control group; being keen if higher parity, if the posture avoids operative birth, if not a long time in the posture; being not keen; ineligibility as planned caesarean; deferring decision to doctors judgement; and choice to participate or opt out. For example:

*‘The static nature of the position would dissuade me from participating in the study.’* (P121, age 26–35 years)

*‘… this is my first pregnancy. I would not prefer to participate in a trial as this is a new experience for me … however I'd be happy to participate if this were my 2nd or 3rd pregnancy …’* (P74, age 16–25 years, Pacific Peoples)

*‘Posterior birth ended in emergency caesarean, and unable to attempt a natural birth for further deliveries.’* (P74, age 16–25 years, Pacific Peoples)

Finally, five respondents provided other comments including a cultural concern that pillows used between the knees to assist the posture should not subsequently be used for the head; that they had heard of yoga postures in pregnancy to prevent malposition; a description of a previous labor and ventouse extraction birth for fetal malposition; and that they were motivated to participate in a future trial (n=2). Examples include:

*‘Having had a long labor when baby was facing the wrong way, I feel quite motivated to get involved with any study that might help me or future women with this!!’* (P11, age 26–35 years, European)

*‘I never heard about this, but if this could help to give birth, I would like to do it.’* (P71, age 26–35 years, Asian)

### Influence of maternal characteristics on trial participation

Respondents identifying as MELAA (n=3; 60%) and European (n=34; 49%) were most likely to indicate they would participate in an RCT of posture for malposition compared to Māori who were equally likely or unsure (each n=6; 43%), and women of Asian or Pacific ethnicity who were most likely to be unsure (n=39; 62% and n=14; 58%, respectively) (p=0.037) (Table 2).

**Table 2:** The influence of maternal characteristics on pregnant women’s likely participation in a future randomized controlled trial of posture for fetal malposition in labor

*Maternal characteristics*	*Yes n (%)*	*Maybe n (%)*	*No n (%)*	*Total n*	*p*
**Age** (years)					0.204*
16–25	4 (36)	4 (36)	3 (27)	11	
26–35	55 (41)	59 (44)	20 (15)	134	
≥36	8 (24)	21 (64)	4 (12)	33	
**Gestation** (weeks)					0.311*
24–28	13 (34)	22 (58)	3 (8)	38	
29–33	24 (43)	25 (45)	7 (13)	56	
34–38	24 (35)	34 (49)	11 (16)	69	
≥39	5 (42)	3 (25)	4 (33)	12	
**Parity** (n=178)					0.338
Nulliparous	35 (37)	43 (45)	18 (19)	96	
Multiparous	31 (38)	42 (51)	9 (11)	82	
**Ethnicity** (n=176)					0.037*
Māori	6 (43)	6 (43)	2 (14)	14	
Pacific people	8 (33)	14 (58)	2 (8)	24	
European	34 (49)	22 (31)	14 (20)	70	
Asian	16 (25)	39 (62)	8 (13)	63	
MELAA^a^	3 (60)	2 (40)	0 (0)	5	
**Experienced malposition** (n=179)					0.844*
Yes	11 (42)	11 (42)	4 (15)	26	
No	56 (37)	74 (48)	23 (15)	153	
**Heard of malposition** (n=179)					0.075
Yes	54 (41)	56 (42)	22 (17)	132	
No	13 (28)	29 (62)	5 (11)	47	
**Heard of using posture** (n=179)					0.506*
Yes	17 (33)	24 (47)	10 (20)	51	
Not sure	5 (29)	11 (65)	1 (6)	17	
No	45 (41)	50 (45)	16 (14)	111	

* Fisher’s exact test.

a Middle Eastern, Latin American, African.

Women who were aware of the use of maternal posture for fetal malposition in labor (n=56; 28%) were less likely to feel the need to consult their partner to decide on trial participation (33%) compared to women who were not aware of the use of posture (54%) (p=0.046).

The acceptability of a future trial of maternal postures for malposition, was not influenced by any other maternal characteristics.

## DISCUSSION

Key findings from the survey of 206 pregnant women planning to birth at a tertiary hospital in Auckland were: the majority of women knew about fetal malposition; were interested in labor research; viewed the Sims posture acceptable and had no cultural, religious, comfort, safety, or other concerns with it; were either unsure or would participate in a future RCT of posture for fetal malposition; and felt the need to consult their partner before consent. Only 28% of women had heard of using posture to correct malposition.

The response rate of 37% reflects the challenges of the COVID-19 pandemic but also a declining consumer survey response rate seen in other studies to 54% in 2008^33^. The ethnicity of all participants closely resembled Auckland Hospital’s population profile^30^ in which European and Asian ethnicities are predominant.

Similar numbers of women were both disappointed and negative compared to satisfied and positive with their previous labor with a fetal malposition, which is of concern when care is aimed at women’s satisfaction and emotional well-being. In this study, it was not possible to elucidate how much a woman’s feelings about the arrival of their baby might confound their expression of a previous malposition in labor. Whether feelings, for example, of joy or gratitude in her baby, limited a woman’s desire to express negative outlooks requires further qualitative research.

The only maternal characteristic assessed that influenced participation in a trial of posture for fetal malposition was ethnicity. Asian and Pacific Peoples were more likely to be unsure about participation. Whether cultural variations in levels of certainty is a factor for research participation generally or whether uncertainty may have been ameliorated with the use of multilingual questionnaires is unclear and requires further research. Women were not specifically asked why they would participate, so comparisons cannot be made with the Cochrane review by Houghton et al.^23^ that assessed factors associated with trial recruitment of pregnant women.

Participation in a future RCT was not influenced by age, gestation, parity, previous experience of malposition, or whether a woman had heard of malposition and the use of posture to correct it. However, the acceptability of the Sims posture was significantly influenced by age, ethnicity, and knowledge of malposition and maternal posture to correct it. Uncertainty about the acceptability of the Sims posture was understandably associated with being unaware of the use of posture, but more specifically being aged ≤26 years, which may reflect less time in years for exposure to more in-depth information related to malposition. Given that 56% of those who were aware of maternal posture to correct malposition derived this knowledge via their midwife or doula/educator suggests midwives and educators could have an impact on participation of a posture trial reflecting what is already known about influencing factors on participation in an RCT^23^.

Comfort concerns and a wish for greater mobility were elucidated from emergent themes on the acceptability of the Sims posture and participation in a future trial. These concerns identify with another study investigating posture exercises in pregnancy for fetal malposition in which the most common reason for ceasing postures was discomfort^24^. Alternatively, subthemes of ‘easier breathing’ in the Sims posture and ‘acceptable if it works’ are possible exemplars why other women thought the posture was acceptable. Considering efficacy of the posture is unknown until after data analysis, the subtheme ‘acceptable if it works’ resonates with a 2021 Cochrane systematic review^23^ of factors impacting RCT recruitment in which women assumed the intervention was likely to be more effective than the control, demonstrating the need for participant information pre-recruitment to clearly explain that efficacy of the intervention is unknown. Given the importance of observing indigenous Māori women’s views concerning future trials^34,35^, it is of note that two women (different ethnicities) referred to the illustrated pillow between the legs. These women indicated they were unsure how acceptable the Sims posture was. Illustrations of the posture may be more acceptable without this optional pillow (normally reserved for the head which is Tapῡ [sacred] in Māori tikanga [culture]).

Prior knowledge of maternal postures to correct malposition was associated with women feeling less need to consult a partner to decide on trial participation, suggesting indecision may relate to the need for greater understanding by women. Decision making regarding participation may be easier if pre-consent discussions about the trial occurred when their partner is present. In many instances, the most likely time when partners are present is in labor. Considering fetal position may change before labor becomes established^36^, it may be prudent to recruit women during established labor once fetal malposition is confirmed and the partner is present rather than antenatally, thereby enhancing recruitment rates as previously reported^27^. However, pre-consent trial enrolment would allow women and their partners to consider participant information in preparation for decision making in labor.

### Strengths and limitations

Strengths of this study include the provision of both quantitative and qualitative data relating to pregnant women’s views on the acceptability of a potential trial of posture for fetal malposition, providing clarity for health professionals and researchers to ensure planned research is relevant. Respondents were at least 24 weeks pregnant enabling women to perceive how uptake of a posture might feel with a gravid uterus to better reflect the trial situation.

However, the survey has some limitations including a potential risk of interpretative bias of thematic content, though this was mitigated using a second non-midwife reviewer. Women who responded to the survey may be more motivated, thus the findings may reflect more positive views than those of non-respondents. Unfortunately, it was not possible for respondents to revise former question responses if they changed their mind during the survey.

Given the hypothetical question regarding future participation in an RCT, and that random trial allocation was explained within the question, it is perhaps not surprising women were slightly more likely to be unsure rather than sure about participation in a future trial. It was promising that relatively few women would not participate. This resonates with the findings by Chamberlain et al.^24^ that women highly valued midwifery research and believed midwives best understood labor and birth. Unlike their study, this study did not collect data on why women would participate in a trial of maternal posture, so conclusions cannot be drawn whether the welfare of the baby was the primary reason for participation.

### Future implications

Barriers towards participation in a future trial of posture for fetal malposition in labor mostly relate to perceived discomfort resulting from restricted movement during labor or safety concerns such as ‘squashing the fetus’. Therefore, the acceptability of such a trial may be enabled through the exploration of any safety concerns with the woman’s antenatal care giver. For example, fears of squashing the fetus may be quelled with explanations that the fetus will move to a more comfortable position accordingly. Optional use of bean bags to support and distribute body weight during use of the intervention posture would enhance comfort and provide reassurance regarding comfort of the fetus. In addition, antenatal education aimed at teaching women how to position their bodies, to allow the forces of gravity to the buoyant fetal trunk to facilitate anterior rotation, needs to occur and may inspire trial participation. Periods of flexibility of movement for the intervention group could lead to greater compliance with the trial protocol. For example, the provision of up to 20 minutes per hour to walk or use alternative postures excepting supine or recumbent postures. Finally, provision of pre-trial information at a time when partners are present will allow more opportunity for informed discussion between the woman and her partner and enable both partners to air their concerns or questions with the maternity care provider. The provision of multilingual pre-trial information may quell uncertainty about trial participation by engendering optimal understanding, as well as cultural respect and inclusivity.

## CONCLUSIONS

The findings from this study provide valuable information of pregnant women’s views about the likelihood of participation in a future trial of maternal posture for fetal malposition in labor. Enablers for trial participation include women learning about fetal malposition, exploring any safety concerns regarding use of maternal postures with their antenatal care giver; periods of flexibility of movement for the trial intervention group; and timing the provision of pre-trial information when partners are present.

## Data Availability

The data supporting this research are available from the authors on reasonable request.
